# CT-based auto-segmentation of multiple target volumes for all-in-one radiotherapy in rectal cancer patients

**DOI:** 10.1186/s13014-025-02694-9

**Published:** 2025-08-19

**Authors:** Xuemin Li, Luqi Wang, Mengying Yang, Xianan Li, Ting Zhao, Mingqing Wang, Siyi Lu, Yunsong Ji, Wei Zhang, Lecheng Jia, Ran Peng, Junjie Wang, Hao Wang

**Affiliations:** 1 Cancer Center, Peking University Third Hospital, Beijing, 100191 China; 2 Department of Radiation Oncology, Peking University Third Hospital, Beijing, 100191 China; 3 Beijing Key Laboratory for Interdisciplinary Research in Gastrointestinal Oncology (BLGO), Peking University Third Hospital, Beijing, 100191 China; 4 Shanghai United Imaging Healthcare Co., Ltd, Shanghai, 201807 China; 5 Department of Radiation Therapy, Peking University People’s Hospital, Beijing, 100044 China; 6 Department of Radiation Oncology, Beijing Hospital, National Center of Gerontology, Institute of Geriatric Medicine, Chinese Academy of Medical Sciences, Beijing, 100005 China; 7 Department of Oncology, Chengyang People’s Hospital, Qingdao, 266109 China

**Keywords:** Rectal cancer, Neoadjuvant radiotherapy, Deep learning, Auto-segmentation, Online adaptive radiotherapy, All-in-One radiotherapy

## Abstract

**Background:**

This study aimed to evaluate the clinical feasibility and performance of CT-based auto-segmentation models integrated into an All-in-One radiotherapy workflow for rectal cancer.

**Methods:**

This study included 312 rectal cancer patients, with 272 used to train three nnU-Net models for CTV45, CTV50, and GTV segmentation, and 40 for evaluation across one internal (*n* = 10), one clinical AIO (*n* = 10), and two external cohorts (*n* = 10 each). Segmentation accuracy (DSC, HD, HD95, ASSD, ASD) and time efficiency were assessed.

**Results:**

In the internal testing set, mean DSC of CTV45, CTV50, and GTV were 0.90, 0.86, and 0.71; HD were 17.08, 25.48, and 79.59 mm; HD 95 were 4.89, 7.33, and 56.49 mm; ASSD were 1.23, 1.90, and 6.69 mm; and ASD were 1.24, 1.58, and 11.61 mm. Auto-segmentation reduced manual delineation time by 63.3–88.3% (*p* < 0.0001). In clinical practice, average DSC of CTV45, CTV50 and GTV were 0.93, 0.88, and 0.78; HD were 13.56, 23.84, and 35.38 mm; HD 95 were 3.33, 6.46, and 21.34 mm; ASSD were 0.78, 1.49, and 3.30 mm; and ASD were 0.74, 1.18, and 2.13 mm. The results from the multi-center testing also showed applicability of these models, since the average DSC of CTV45 and GTV were 0.84 and 0.80 respectively.

**Conclusions:**

The models demonstrated high accuracy and clinical utility, effectively streamlining target volume delineation and reducing manual workload in routine practice.

**Trial registration:**

The study protocol was approved by the Institutional Review Board of Peking University Third Hospital (Approval No. (2024) Medical Ethics Review No. 182-01).

## Introduction

Colorectal cancer is the third most prevalent cancer worldwide [1] with rectal cancer accounting for approximately one-third of these cases. Radiotherapy is a crucial treatment modality for the majority of patients diagnosed with rectal cancer, which can significantly improve local-regional control (LRC) rates of tumors and enhance overall survival (OS) [2–5]. Traditional radiotherapy workflows often require 1–2 weeks from imaging to treatment. In contrast, All-in-One (AIO) radiotherapy can condense simulation, planning, and delivery into a single session—typically within 30 min—thereby streamlining care delivery and minimizing treatment delays [6].

The AIO workflow integrates simulation, contouring, planning, image guidance, beam delivery, and in vivo QA using the uRT-linac 506c (United Imaging Healthcare, Shanghai), a CT-integrated linac with a 16-slice helical CT scanner. A proprietary software suite (uRT-TPS and TDS) manages treatment planning, delivery, and a shared patient database. More information about the uRT-linac 506c platform can be found in a previous report [7].

Contouring is a key step in the AIO process and is done by the artificial intelligence (AI) modules in the uRT-TPS, since the general medical images segmentation models rarely satisfied the specific needs of our department. Deep Learning (DL) methods are the state-of-the-art approach for tackling automated medical image segmentation tasks, with the UNet [8] being the most widely adopted network variation [9]. Currently, the literature based on DL medical image segmentation focuses predominantly on network architecture and architectural modifications, such as the integration of residual, dense, or inception blocks, for achieving performance improvements with evaluation commonly conducted on a single dataset or restricted number of datasets [10–18].

The nnU-Net framework has been developed by Isensee et al. [19] and can adapt for any segmentation task, self-configures the network training pipeline while considering computer-hardware capabilities and dataset specific properties. The systematic pipeline selection enables nnU-Net to achieve top segmentation performance in 33 of 53 anatomical structures, matching expert-level accuracy. Its self-configuring U-Net architecture allows integration of advanced modifications for optimization. DL models, trained on departmental clinical data, were expert-evaluated and integrated into uRT-TPS for clinical use.

In this study, we aimed to develop and validate deep learning-based auto-segmentation models, specifically using the nnU-Net framework, for delineating multiple target volumes in rectal cancer radiotherapy. The clinical applicability of these models was assessed across internal, real-world AIO, and multi-center datasets.

## Materials and methods

### Data collection

#### Patient cohort

**Fig. 1:**
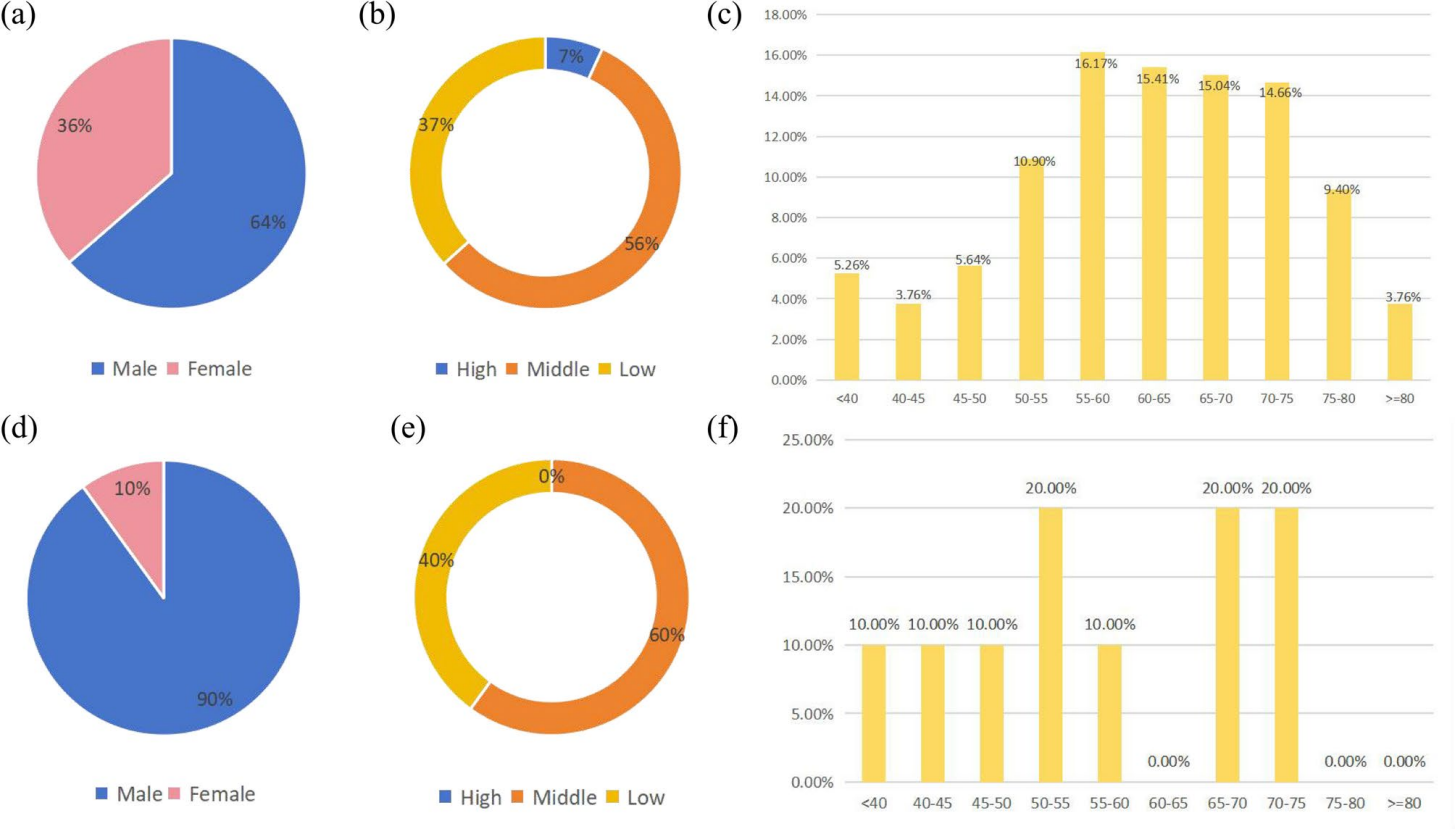
(**a**)-(**c**) Clinical data of patients in the training cohort. **(d**)-(**f**) Clinical data of patients in the internal testing cohort. (**a**) and (**d**) show the distribution of male and female patients; (**b**) and (**e**) indicate the locations of the target; (**c**) and (**f**) illustrate the age distribution

This study was approved by our institutional review board (Peking University Third Hospital). A total of 312 patients with rectal cancer were included. Among them, 282 patients with T_3 − 4_ stage rectal cancer treated at our institution between June 2012 and June 2024 were included in this work. The 282 retrospective data were categorized into training (272/282) and internal testing groups (10/282) by random sampling (Fisher-Yates shuffle). The patients in training cohort received neoadjuvant radiotherapy, the standard regime for locally advanced high-low rectal cancer. Among the patients, 64% of the total patient cohort were male and 36% were female (Fig. 1a), and the ages ranged from 22 to 97 years old with a median age of 62 years. In the internal testing cohort, 90% of the patients were male and 10% were female (Fig. 1d). The ages ranged from 35 to 74 years, with a median age of 55 years. There were no patients with high-rectal cancer in the testing cohort. The distribution of the location of the target, age, and gender in the testing set closely aligned with that in the training set and remained within the same range. This consistency further supports the scientific rigor of the study.

In addition, a cohort of 10 T_3 − 4_ stage rectal cancer patients, treated at our institution between May and June 2024 with AIO radiotherapy, was used to evaluate the segmentation performance of the models. And 20 external cases from two independent centers were used for external validation.

#### Image acquisition and data Preparation

All the patients underwent RT-specific plain and contrast CT scans (CT simulations) in the supine posture. The CT images were acquired on a big-bore RT-specific CT scanner (Brilliance Bigbore CT, Philips, Netherlands) with 5-mm slice thickness. The axial image size was 512 × 512 with a spatial resolution of 1.172 mm × 1.172 mm. The plain CT and contrast CT images were scanned at the same time and rigidly registered on the uTPS (u-Treatment Planning System, United Imaging Inc., China) by a physicist, and afterwards CTV45, CTV50 and GTV volumes were delineated on CT images by an oncologist.

Paired CT images, spatial transformations, and target contours for all patients were de-identified and used for model training and evaluation. Retrospective datasets were reviewed by an oncologist panel to serve as the ground truth, while prospective AIO patient data were included for real-world validation.

### Model description

We used the 3D version of the publicly available nnU-Net framework [19] to develop segmentation models for CTV45, CTV50, and GTV based on planning CT images. This self-configuring deep learning framework automatically adapts its architecture, preprocessing, training, and post-processing parameters according to dataset characteristics and available computational resources. Each model was trained using a 3D U-Net backbone with deep supervision. The loss function combined Dice and cross-entropy loss, and training was performed using the ADAM optimizer [20] with default nnU-Net settings. Data augmentation strategies were applied automatically during training.

The nnU-Net framework utilizes a 3D U-Net architecture with skip connections, enabling feature maps from the encoder to aid in the decoder’s reconstruction. It employs 3D convolutions for feature extraction, transposed convolutions for up-sampling, and strided convolutions for down-sampling. Furthermore, the network utilizes deep supervision [21] in all but the two deepest levels to improve gradient flow.

### Evaluation of model performance and time efficiency

#### Internal testing of the models

The internal testing set, comprising CT simulation images (plain CT) from 10 patients, was used to evaluate model performance and time efficiency. Firstly, the trained model was applied to automatically generate three contours (CTV45, CTV50 and GTV) for the 10 patients. Secondly, two senior oncologists (with ≥ 5-year clinical experience) were invited to delineate CTV45, CTV50 and GTV on the plain CT images manually without preview for assessing time cost. Finally, the time efficiency of auto-segmentation and manual delineation was compared, and their similarity was quantitatively assessed using standard evaluation metrics, including the Dice Similarity Coefficient (DSC), Hausdorff Distance (HD), 95th percentile Hausdorff Distance (HD 95), Average Surface Distance (ASD), and Average Symmetric Surface Distance (ASSD).

The two oncologists involved in the evaluation phase were not involved in annotating the training data, thereby ensuring independence between the training and testing processes.

#### **Clinicalapplication in AIO radiotherapy and multi-center testing of the models**

To assess clinical applicability, we evaluated the effectiveness of multiple auto-segmentation models in 10 rectal cancer patients undergoing AIO radiotherapy. All patients had T_3 − 4_ stage disease, underwent neoadjuvant radiotherapy, and received AIO treatment using a CT-linac platform. Quantitative indicators were also calculated.

Two external datasets (10 patients each) were used for multi-center testing, with the same indicators used to evaluate the model’s compatibility and robustness. These datasets were retrospectively collected and included only patients with T_3 − 4_ stage rectal cancer who underwent neoadjuvant therapy. Manual contours for CTV45 were reviewed and standardized by our expert team prior to evaluation to ensure consistency.

GTV annotations from center B were excluded due to incomplete coverage (only superior and inferior axial slices), which reflects local contouring practice. However, CTV annotations were complete and included in the analysis.

### Statistical analysis

Data management and statistical analyses were conducted using Microsoft Excel™ (MS Office 365) and SPSS version 26.0. Auto-segmentation outcomes were evaluated with metrics such as DSC, HD, HD 95, ASD, and ASSD. Data are presented as mean ± standard deviation unless stated otherwise. The time required for automated segmentation and oncologist modifications was compared to that of manual delineation. Normality and variance assumptions were checked; if met, an independent samples t-test was applied. Non-parametric tests (Mann-Whitney U test) were used for data with unequal variances. Statistical significance was set at *p* < 0.05.

## Results

### Visualization of representative cases

**Fig. 2:**
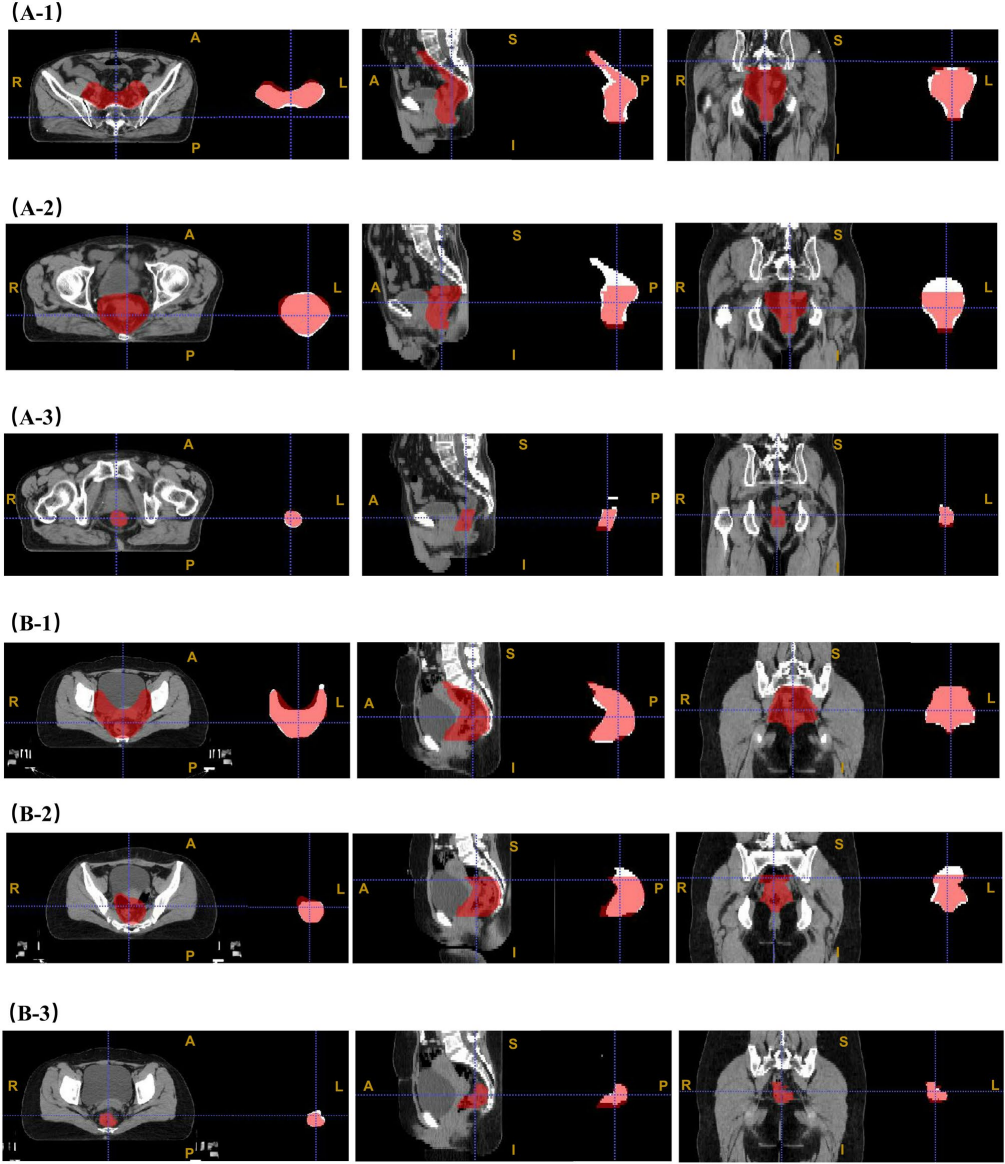
Example visualizations of the datasets at level of 40 and window of 350. Red areas represent manual delineations; white areas represent auto-segmented regions. (**A-1**) to (**A-3**) show patient A’s results for CTV45, CTV50, and GTV, (**B-1**) to (**B-3**) show the same for patient **B**

Figure 2 illustrates two representative cases for visualization. Automated segmentation and manual delineation of CTV45, CTV50 and GTV are shown for case A and case B across transverse, sagittal, and coronal planes, respectively. The auto-segmentation models demonstrated performance closely matching manual delineation, requiring only minor adjustments.

### Segmentation accuracy analysis

The DSC, HD, HD 95, ASSD and ASD of the automated segmentation outcomes for the ten patients in the internal testing set were summarized in Table 1. Manual delineation and automated segmentation showed good agreement for CTV45 and CTV50, with the average DSC for both reaching ≥ 0.8. Minor manual modifications were needed for the auto-segmented CTV45, CTV50 and GTV regions.

**Table 1:** The value of DSC, HD, HD 95, ASSD and ASD for CTV45, CTV50 and GTV regions in internal testing set. Average score and standard deviation are presented

Target	DSC	HD (mm)	HD 95 (mm)	ASSD (mm)	ASD (mm)
CTV45	0.90 ± 0.04	17.08 ± 7.11	4.89 ± 2.53	1.23 ± 0.43	1.24 ± 0.60
CTV50	0.86 ± 0.06	25.48 ± 14.17	7.33 ± 4.94	1.90 ± 0.86	1.58 ± 0.87
GTV	0.71 ± 0.26	79.59 ± 138.88	56.49 ± 129.31	6.69 ± 11.14	11.61 ± 22.50

### Time efficiency analysis

**Fig. 3:**
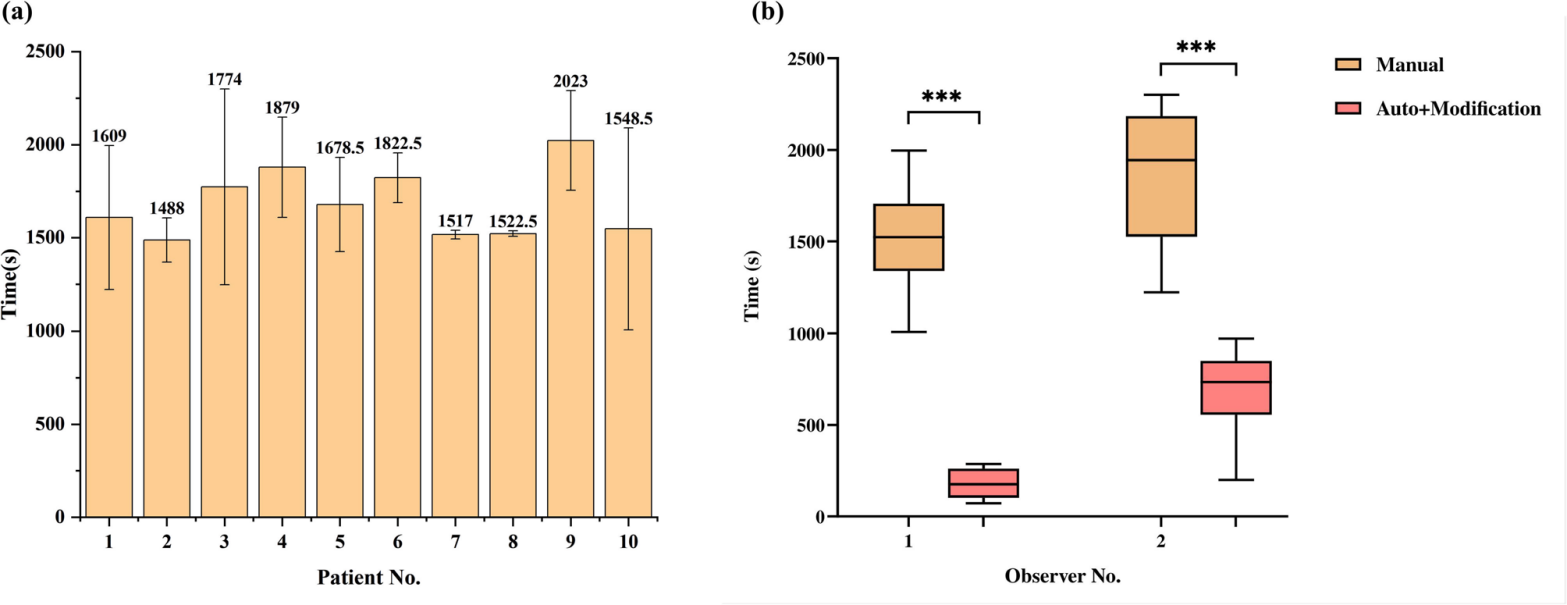
(**a**) Time cost of manual delineation for the ten patients in testing set. Average values and [minimum–maximum] ranges are presented. (**b**) Comparison of time cost between manual delineation and automated segmentation with modification

The time required for manual delineation by two oncologists for the ten patients in testing set are shown in Fig. 3(a), with a mean segmentation time of 1686.2 s per patient.

Comparison of time cost between manual delineation and automated segmentation with modification are shown in Fig. 3(b). The time required for automated segmentation with manual modification was significantly lower than that for manual delineation (Oncologist A: (13.4 + 164.0) seconds vs. 1514.7 s; Oncologist B: (11.4 + 670.9) seconds vs. 1857.7 s) (*p* < 0.0001). Due to differences in experience, the two oncologists exhibited variations in manual delineation and modification time costs. The increased efficiency reduced contouring time by 63.3–88.3%, alleviating workload and minimizing inter-observer variability.

### Clinical application in AIO radiotherapy

**Fig. 4:**
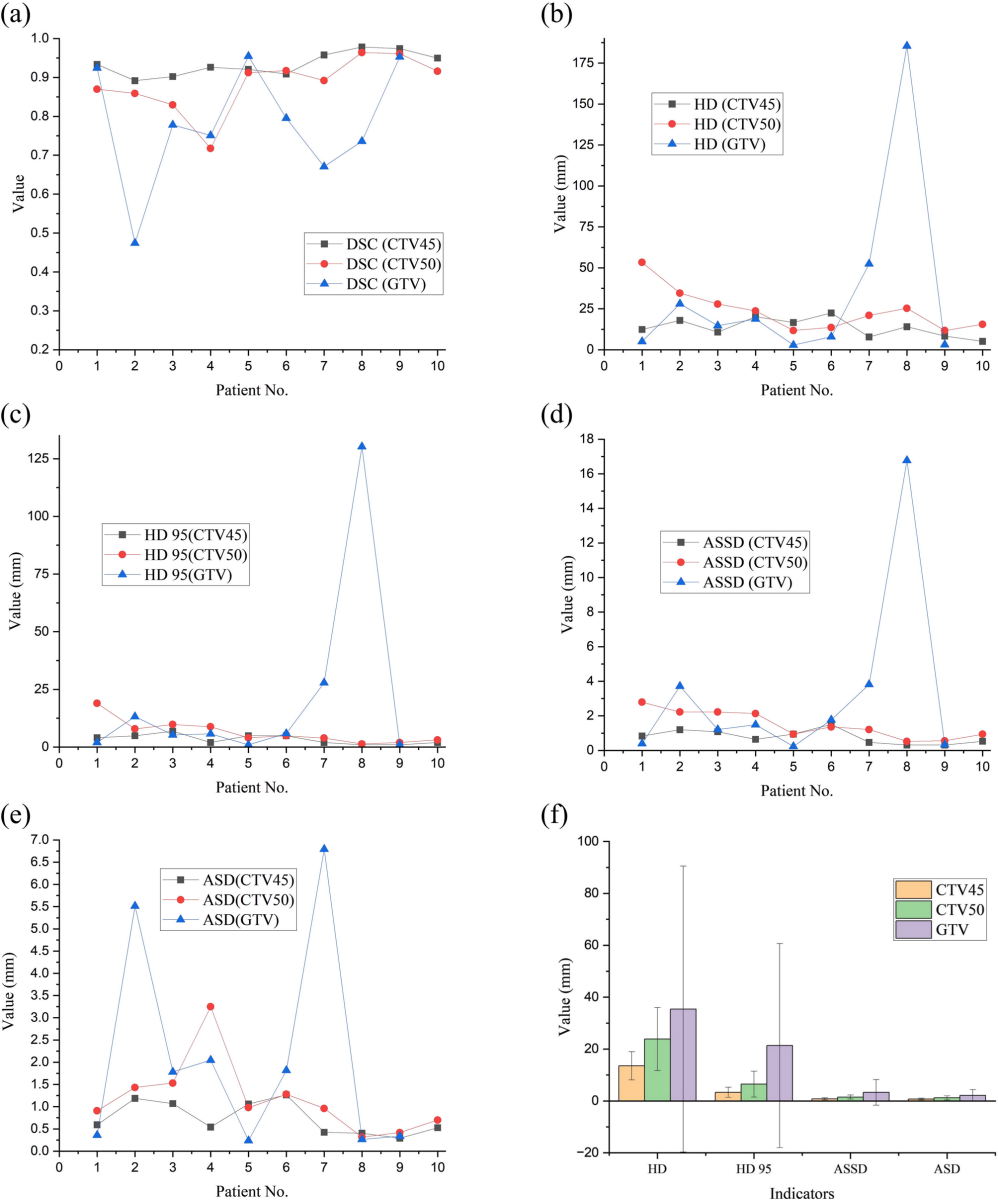
DSC (**a**), HD (**b**), HD 95 (**c**), ASSD (**d**) and ASD (**e**) of the automated segmentation results in ten AIO radiotherapy patients. (**f**) shows the average and STD of HD, HD 95, ASSD and ASD

We evaluated the auto-segmentation models in 10 rectal cancer patients undergoing AIO radiotherapy. Figure 4 presents the DSC, HD, HD 95, ASSD, and ASD metrics. While GTV segmentation showed variability, overall performance remained clinically acceptable. There was a strong correlation between the automated segmentation and the manual delineation. The average DSC of CTV45, CTV50 and GTV were 0.93 ± 0.03, 0.88 ± 0.07, and 0.78 ± 0.15; the average HD were 13.56 ± 5.41, 23.84 ± 12.15, and 35.38 ± 55.12 mm; the average HD 95 were 3.33 ± 1.92, 6.46 ± 4.98, and 21.34 ± 39.33 mm; the average ASSD were 0.78 ± 0.38, 1.49 ± 0.76, and 3.30 ± 4.93 mm; and the average ASD were 0.74 ± 0.35, 1.18 ± 0.79, and 2.13 ± 2.28 mm. In clinical practice, the multiple auto-segmentation models demonstrated excellent accuracy.

### Multi-center testing of models

We also collected additional two sets of data from rectal cancers’ patients from another two hospitals (10 patients per dataset), to evaluate the models’ compatibility and robustness across institutions.

CTV50 delineation was specific to our institution and was not available at the two external centers. Therefore, only GTV and CTV45 models were tested in this experiment. The testing results are shown in Table 2. Except for the CTV50 model, the average DSC for CTV45 and GTV were above 0.80, indicating the clinical applicability in these two centers.

**Table 2:** The multi-center testing results of GTV and CTV45 model. Note that “CTV45-1” and “CTV45-2” were results from two different centers. Average score and standard deviation are presented

Model	DSC	HD (mm)	HD 95 (mm)	ASSD (mm)	ASD (mm)
CTV45-1	0.84 ± 0.02	101.04 ± 225.71	6.95 ± 1.35	2.16 ± 0.46	2.00 ± 0.91
CTV45-2	0.85 ± 0.04	30.79 ± 16.69	8.00 ± 1.70	2.18 ± 0.45	2.21 ± 0.61
CTV50	/	/	/	/	/
GTV	0.80 ± 0.09	21.59 ± 18.23	5.35 ± 2.65	1.67 ± 0.71	1.37 ± 0.69

**Fig. 5:**
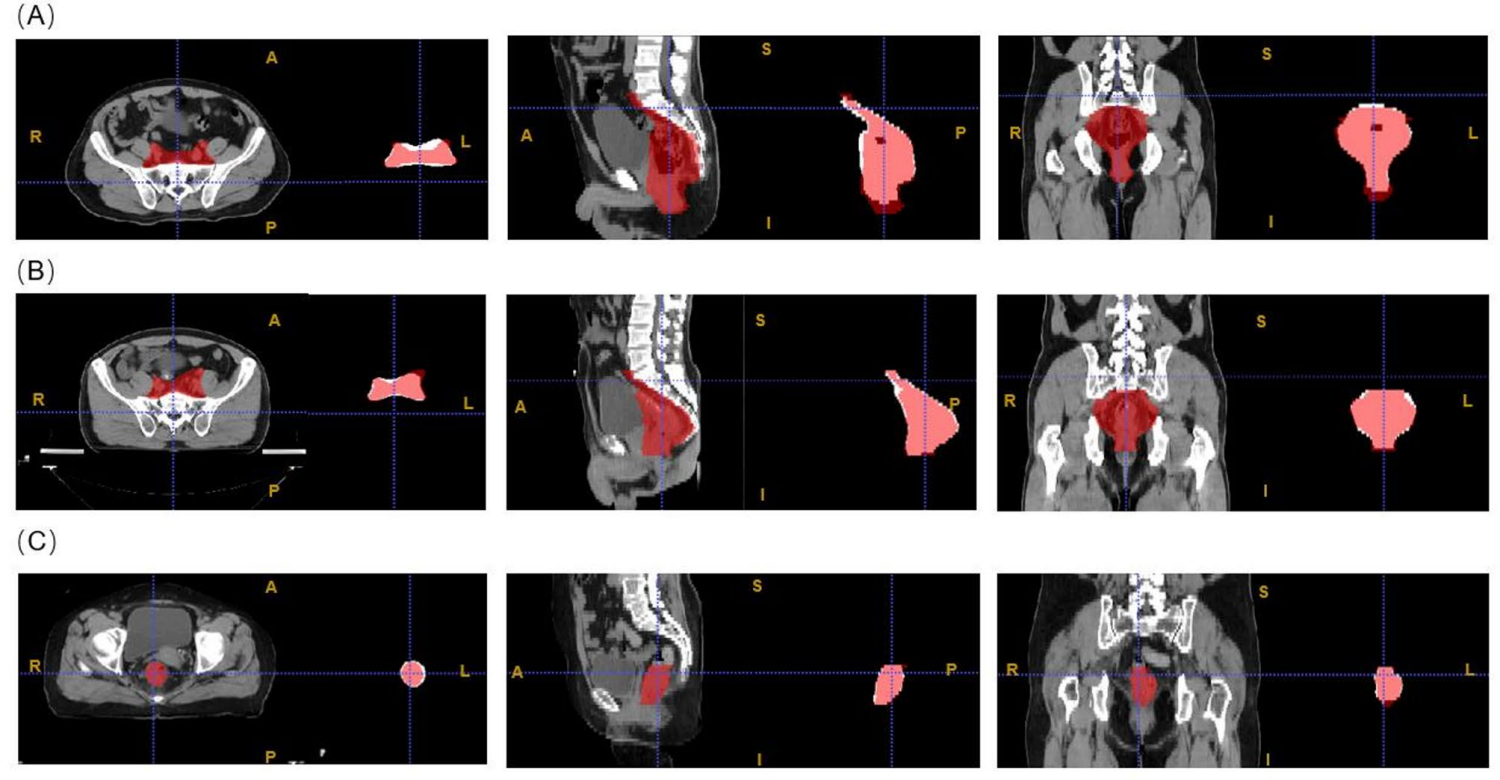
Example visualizations of the multi center testing datasets at level of 40 and window of 350. In each subfigure, red area represents manual delineation region, white area represents auto-segmentation region. Figure (**A**) is the testing results of CTV45 model from center A, Figure (**B**) is the testing results of CTV45 model from center B, Figure (**C**) is the testing results of GTV model from center A

As shown in Fig. 5, CTV45 and GTV auto-segmentation aligned well with manual contours. However, GTV contours from center B included only the top and bottom layers, leading to significant discrepancies. Thus, center B’s GTV auto-segmentations were excluded.

## Discussion

### Clinical benefits from multiple target volumes auto-segmentation models

The CT-based multiple target volumes auto-segmentation models were optimized based on the nnU-Net framework using CT data from our center. The localized models are better adapted to the individualized patient characteristics at our institution. Deep learning models, especially using CNNs, can significantly enhance the efficiency of target volumes segmentation for rectal cancer patients [22, 23]. For rectal cancer target segmentation, manual delineation takes around 30 min, whereas deep learning-based auto-segmentation completes in seconds. With oncologist modifications, the total time reduces to 3–11 min, alleviating workload and minimizing errors. These models efficiently process large-scale CT data, enhancing workflow in both AIO and conventional radiotherapy. Although the observed total segmentation time may seem modest, reducing delay is crucial in time-sensitive workflows like pelvic AIO radiotherapy, where bladder filling may alter anatomy. Notably, current literature rarely quantifies manual editing time, and future studies should evaluate the full “AI + human-in-the-loop” efficiency to inform clinically deployable models.

Our auto-segmentation models achieved high precision, with DSC for CTV exceeding 0.8. Other metrics also showed strong performance, aligning closely with manual delineation and meeting clinical accuracy requirements. By standardizing segmentation, these models minimize subjective errors and enhance consistency. The integrated AIO workflow reduces systematic uncertainties between simulation and treatment. The impact of systematic changes in patient anatomy, such as tumor progression and weight fluctuations after the simulation, is minimized due to the accelerated treatment preparation process [6]. The proposed auto-segmentation models demonstrated strong segmentation accuracy and time efficiency, highlighting their potential to streamline radiotherapy workflows and alleviate the burden of manual contouring.

In clinical practice, the accuracy of the auto-segmentation models has been further validated. The results of automated and manual delineation demonstrated a strong similarity in the CTV, as evidenced by the high DSC. Additionally, the HD, HD 95, ASSD, and ASD metrics further indicated high segmentation accuracy in the CTV. Several previous studies have evaluated deep learning–based auto-segmentation models for CTV delineation in rectal cancer, with reported mean DSC values typically ranging from 0.817 to 0.96, depending on model architecture (such as Flex U-Net, DeepLabv3+, and DDCNN) and cohort size [23–26]. Our model showed comparable or superior accuracy, achieving mean DSCs of 0.90 for CTV45 and 0.86 for CTV50 on the internal testing set, and up to 0.93 and 0.85, respectively, in the clinical AIO and external cohorts. These results underscore the robustness of our model and its suitability for clinical integration.

Overall, the CT-based auto-segmentation models demonstrated the potential to improve the efficiency, consistency, and accuracy of target volume segmentation in rectal cancer AIO radiotherapy, thereby highlighting their superior clinical applicability. While the segmentation time was significantly reduced, we acknowledge that this metric alone does not reflect clinical usability. Future work will focus on systematically quantifying manual correction effort and clinical acceptability to complement speed improvements. In addition, we are prospectively evaluating the feasibility of a fully automated, zero-intervention workflow and identifying patient cohorts for whom such an approach may be suitable.

### Multi-center validation and generalizability of our models

To further evaluate the generalizability of our auto-segmentation models, we performed multi-center validation using datasets from two additional hospitals. The results demonstrated that the DSC values for CTV45 and GTV consistently exceeded 0.80, confirming the models’ high accuracy across diverse clinical settings. However, variations in target volume delineation conventions were observed among institutions, particularly regarding the definitions of CTV45 and CTV50.

At our institution, a dual-prescription approach is employed, wherein CTV45 and CTV50 are separately delineated, with distinct dose levels assigned to the mesorectal and nodal regions. In contrast, many other centers utilize a single-prescription approach, in which the entire CTV is uniformly treated at a single dose level. In these institutions, the CTV is functionally equivalent to our CTV45, without a distinct definition for CTV50. Consequently, in our multi-center validation, external centers’ CTVs were considered equivalent to our CTV45 for comparative analysis. This distinction is essential for accurately interpreting the multi-center validation results and clarifies why CTV50 was not included in the external validation process.

The auto-segmentation models demonstrated reduced accuracy for GTV in some cases. Notably, performance was lower in the internal testing set (DSC = 0.71 ± 0.26), compared to the clinical AIO cohort (DSC = 0.78 ± 0.15) and the external center set (DSC = 0.80 ± 0.09), indicating a need for further investigation. The internal testing set, based on retrospective data from 2012 to 2024, showed greater variability in imaging protocols, tumor morphology, and contouring quality, including small and irregular tumors that are inherently difficult to segment [27, 28]. In contrast, both the clinical AIO and external center cohorts achieved higher DSC and more stable results. This may reflect standardized imaging workflows, better-defined tumors, and consistent annotations—especially in the external sets, which were curated or reviewed by the training team [6]. The boundaries of the CTV are generally easier to identify, leading to better segmentation performance.

### Limitations

One key limitation is the small sample size used for model training and internal testing. Although care was taken to match the demographic and anatomical distributions with the training cohort, we acknowledge that the relatively limited data in testing cohorts may limit statistical robustness. Future work will incorporate repeated sampling or cross-validation to enhance the generalizability of performance estimates. Another limitation of the present study is the lack of detailed clinical and anatomical characterization of the patient population. Variables such as tumor volume, nodal status, prior treatments, and pelvic morphology may influence segmentation performance and should be considered in future validation efforts.

In addition, the GTV segmentation results from one external center (center B) were excluded due to incomplete ground truth contours (limited to superior and inferior axial slices), although the corresponding CTV annotations were retained. This may slightly limit the comprehensiveness of the external validation.

Future clinical applications should incorporate more patient data to optimize the models, mitigate overfitting, and improve their robustness in real-world settings. To support this, we are currently building a prospective registry of AIO-treated rectal cancer patients at our center, which includes detailed clinical metadata (e.g., tumor location, volume, stage, prior therapy). This resource will enable more granular subgroup analysis and allow evaluation of model performance across diverse clinical conditions.

Additionally, the development and deployment of the AIO workflow are constrained by the availability of resources, including both financial and human capital. The acquisition of the necessary equipment, the CT-linac combination and the high demands on the experience and expertise of oncologists and medical physicists, presents significant barriers to the widespread adoption of AIO radiotherapy in many other centers. Future research should concentrate on investigating weakly-supervised learning methodologies, as these approaches can diminish dependence on specialists and enhance the accessibility and scalability of the AIO workflow in clinical practice.

## Conclusion

CT-based auto-segmentation models demonstrated high accuracy and time efficiency in rectal cancer radiotherapy. Multi-center validation confirmed the robustness of our models and highlighted variations in CTV delineation practices across institutions. Future research should focus on improving dataset diversity and model adaptability to facilitate broader clinical integration. These findings support the clinical readiness of our models and their potential for broader translational application in real-world AIO workflows.

## Data Availability

No datasets were generated or analysed during the current study.

## Declarations

### Authors’ information

Corresponding author information: Dr. Hao Wang is a Chief Physician, Associate Professor, and PhD Supervisor at the Cancer Center of Peking University Third Hospital. She is recognized as an Outstanding Doctor, a Key Young Academic Member, and a back-up candidate for Academic and Technical Leadership. Her clinical and research interests focus on radiotherapy for gastrointestinal, urological, and lymphatic malignancies.

## Abbreviations

LRC: local-regional control
OS: overall survival
AIO: All-in-One
QA: quality assurance
UIH: United Imaging Healthcare
TDS: treatment delivery system
CNN: convolutional neural network
DL: Deep Learning
DSC: Dice Similarity Coefficient
HD: Hausdorff Distance
HD 95: 95% Hausdorff Distance
ASSD: Average Symmetric Surface Distance
ASD: Average Surface Distance
CTV: Clinical Target Volume
GTV: Gross Tumor Volume
